# The Impacts of Cardiac Rehabilitation Program on Echocardiographic Parameters in Coronary Artery Disease Patients with Left Ventricular Dysfunction

**DOI:** 10.1155/2013/201713

**Published:** 2013-12-29

**Authors:** Masoumeh Sadeghi, Mohammad Garakyaraghi, Mohsen Khosravi, Mahboobeh Taghavi, Nizal Sarrafzadegan, Hamidreza Roohafza

**Affiliations:** ^1^Cardiac Rehabilitation Research Center, Isfahan Cardiovascular Research Institute, Isfahan University of Medical Sciences, Isfahan 81465-1148, Iran; ^2^Heart Failure Research Center, Isfahan Cardiovascular Research Institute, Isfahan University of Medical Sciences, Isfahan 81465-1148, Iran; ^3^Isfahan Cardiovascular Research Center, Isfahan Cardiovascular Research Institute, Isfahan University of Medical Sciences, Isfahan 81465-1148, Iran; ^4^Psychosomatic Research Center, Isfahan Cardiovascular Research Institute, Isfahan University of Medical Sciences, Isfahan 81465-1148, Iran

## Abstract

*Introduction*. The accurate impact of exercise on coronary artery disease (CAD) patients with left ventricular dysfunction is still debatable. We studied the effects of cardiac rehabilitation (CR) on echocardiography parameters in CAD patients with ventricular dysfunction. *Methods*. Patients with CAD who had ventricular dysfunction were included into an exercise-based rehabilitation program and received rehabilitation for eight weeks. All subjects underwent echocardiography before and at the end of the rehabilitation program. The echocardiography parameters, including left ventricular ejection fraction (LVEF), LV end-diastolic (LVEDD) and end-systolic diameters (LVESD), and peak exercise capacity measured in metabolic equivalents (METs), were assessed. *Results*. Seventy patients (mean age = 57.5 ± 10.2 years, 77.1% males) were included into the study. At the end of rehabilitation period, the LVEF increased from 45.14 ± 5.77% to 50.44 ± 8.70% (*P* < 0.001), and the peak exercise capacity increased from 8.00 ± 2.56 to 10.08 ± 3.00 METs (*P* < 0.001). There was no significant change in LVEDD (54.63 ± 12.96 to 53.86 ± 8.95 mm, *P* = 0.529) or in LVESD (38.91 ± 10.83 to 38.09 ± 9.04 mm, *P* = 0.378) after rehabilitation. *Conclusion*. Exercise training in postmyocardial infarction patients with ventricular dysfunction could have beneficial effects on cardiac function without adversely affecting LV remodeling or causing serious cardiac complications.

## 1. Introduction

Coronary artery diseases (CAD) are the leading cause of mortality in elderly individuals in developing countries. They account for nearly 50 percent of all deaths per year in Iran [1]. Also, they cause significant morbidity and impair the patient's quality of life [2, 3]. Various echocardiographic parameters have been shown to provide cardiac dysfunction in CAD patients, such as left ventricular volumes and ejection fraction which are strongly related to prognosis of cardiac diseases [4]. Cardiac rehabilitation (CR) is an acceptable treatment strategy adding to the basic medical plan for the patients with CAD. A multifactorial rehabilitation program includes six basic cores which are (1) baseline patient assessment, (2) nutritional counseling and weight management, (3) aggressive coronary risk-factor management, (4) psychosocial management, (5) physical activity counseling, and (6) exercise training. Several studies showed the beneficial effects of CR for CAD patients [5]. According to previous meta-analyses on the effects of exercise-based rehabilitation in patients with CAD, a reduction in total and cardiac mortality and morbidity occurred after CR [6–8]. In this regard, a controlled study on patients with myocardial infarction (MI) with reduced ventricular function treated with cardiac exercised base rehabilitation revealed that oxygen uptake increased by the exercise, but end-diastolic and end-systolic myocardial wall thickness yielded no significant change indicating that the training program had no deleterious effects on left ventricular (LV) volume, function, or wall thickness [9]. Additionally, there are studies that indicate no adverse changes in left ventricle remodeling in patients with ejection fraction of more than 50% [10]. However, in another study, Jugdutt et al. followed the patients after acute MI that had underwent 12 weeks CR program and found that further cardiac functional and topographic deteriorations occur after exercise programs in patients with more severe LV asynergy [11]. The accurate impact of exercise in patients with advanced ventricular dysfunction seems to be debatable. We hypothesized that exercise-based cardiac rehabilitation programs improve echocardiographic parameters in patients with LV dysfunction. Therefore, we aimed to study the effect of rehabilitation program on the echocardiography parameters of CAD patients who had LV dysfunction.

## 2. Methods and Materials

### 2.1. Patients and Settings

This self-controlled trial was conducted on CAD patients referring to Isfahan Cardiovascular Research Center, Isfahan, Iran, between 2011 and 2012. Adult patients after coronary artery bypass grafting (CABG), percutaneous coronary angioplasty (PCA), or MI, with New York Heart Association classes II and III and ejection fraction between 30% and 50% and with LV dysfunction, were included into the study. The patients started the rehabilitation program one month after CABG and MI and two months after PCA. Patients with severe ventricular dysfunction (ejection fraction < 30%), unstable cardiac symptoms, change in medication within the preceding three months, recurrent ischemia, concurrent pulmonary disease, uncontrolled arrhythmia, or sever musculoskeletal disease were not included to the study. Also patients who had taken part in less than half of the rehabilitation sessions or had poor echo window were excluded from the study. The study was approved by the Ethics Committee of the Isfahan University of Medical Sciences and all patients signed a written inform consent before entering the study.

### 2.2. Rehabilitation Program

The rehabilitation program consisted of 20 sessions, scheduled over 8 weeks, 2 to 3 times per week. All participants follow both ergometer and treadmill. They allocate a predefined time in each exercise modality. Each session was about 1.5 hour; the first 10–20 minutes began with a warm-up followed by 20–40 minutes of aerobic exercise and finished with a 10-minute cool down; the patients had 20-minute relaxation at the end of each session [11]. Each session consists of at least 30 minutes aerobic exercise including about 20 minutes treadmill and 10 minutes ergoline cycling. In this part heart monitoring and control of blood pressure have been done. Patients had stretch activities for warm-up and cool-down phases. The intensity of the exercise was calculated according to the determined risk (patients age, underlying disease severity, and exercise test result) [12, 13], between 60 and 85% of the maximum heart rate (HR) according to Naughton protocol achieved on the basic exercise test [14]. Also the load and velocity of the exercise increased during the sessions. According to patients' underlying disease and capacity and the result of exercise test, we increase gradually both load and velocity. In the first three sessions the patients had heart monitoring supervised by a cardiologist. All patients received psychological, nutritional, and smoking cessation consult. In addition, weekly educational sessions were held during the eight weeks of comprehensive rehabilitation program, both for patients and their families. These consisted of explanations on cardiovascular diseases, its risk factors, diagnoses and treatment approaches, medications with their complications, stress reduction methods, and advice on a healthy lifestyle including smoking cessation, nutrition, and physical activity.

### 2.3. Assessments

At baseline and at the end of the rehabilitation period, cardiac peak exercise capacity measured in metabolic equivalents (METs) was evaluated and Doppler-echocardiography was performed. Standard views, including the parasternal long-axis, short-axis at the papillary muscle level, and apical 4- and 2-chamber views were recorded. Left ventricular ejection fraction (LVEF) and end-systolic and end-diastolic diameters (LVESD and LVEDD) were measured according to Simpsons model. The Doppler-echocardiographic studies were all performed by the same cardiologist who was blinded to the study. Also, the patients' demographic date, medical history, and presence of heart failure were recorded.

### 2.4. Statistical Analysis

The data were analyzed using the SPSS software (version 16.0) for windows. Quantitative and qualitative variables are presented as mean ± SD and number (%), respectively. Paired *t*-test was used for comparing LVEF, end-systolic/diastolic diameters, and the peak exercise capacities before and after the treatment. A *P* value of <0.05 was considered significant in all analyses.

## 3. Results

During the study period, 140 patients enrolled to the rehabilitation program from which 84 patients had EF between 30% and 50%. A total of 82 patients agreed and enrolled to the study and 70 patients completely attended the rehabilitation sessions (mean age = 57.5 ± 10.2 years, 77.1% males). Demographic data of the patients are presented in Table 1. All the patients were taking aspirin, beta blockers, and statins. Also, 30 patients had heart failure.

At the end of the rehabilitation period, the LVEF increased from 45.14 ± 5.77% to 50.44 ± 8.70% (*P* < 0.001) and the peak exercise capacity increased from 8.00 ± 2.56 to 10.08 ± 3.00 METs (*P* < 0.001). But no significant change was observed in LVEDD or LVESD after rehabilitation (*P* > 0.05), and the patients had no cardiac complications (Table 2).

## 4. Discussion

The epidemic of our era is CAD and it is estimated to be the single most important disease in the world in the terms of mortality, morbidity, disability, and economy even until 2020. Therefore cardiac preventive programs are the special need of our century. The meta-analysis evaluating the trials on coronary patients treated with exercise-based rehabilitation concluded that all of the coronary risk factors were improved and recurrent MI was reduced by about 20 percent after a year of rehabilitation program, also the mortality rate was reduced with longer follow-up after taking part in the programs [6, 15]. However the effect of CR on ventricular remodeling especially in patients with lower ejection fraction is still debatable [10]. This study originates in Iran, which is a highly understudied population in cardiac rehabilitation. Given the increasing prevalence of CAD and noncommunicable diseases worldwide, this study was needed to document the efficacy of cardiac rehabilitation in Iranian population. In our study, we applied rehabilitation program for CAD patients with ventricular dysfunction. Our study population had the ejection fraction of about less than 50% before entering into the study that was improved significantly after rehabilitation. Moreover, peak exercise capacity was significantly improved in our patients. Also, LVESD and LVEDD had no clinical or statistical change after the program. These results show that, among the patients with LV dysfunction, exercise-based rehabilitation is beneficial and has no detrimental effects on ventricular remodeling.

In rehabilitation programs several techniques are indicated for blood pressure control, smoking cessation, lipid lowering, diabetes and obesity control, and lifestyle modification. Although exercise training affects synthesize of free radicals, it increases the work capacity without a concomitant increase in free radical production. This fact indicates that physical activity could be performed with less oxidative stress. Also physical training reduced insulin resistance in post-MI patients with hyperinsulinemia and homocysteine level in patients with normal lipid profile which reduce the CAD risk for 20 to 30%. In this regard the review indicated that fibrinolysis improved as well as myocardial perfusion after physical training and improves systolic function and ejection fraction by increasing the muscle strength due to increasing heart rate during sympathic states caused by exercise [16].

Our results are consistent with the studies on patients with ejection fraction greater than 50% which indicated that exercise-based rehabilitation program does not have adverse impact on LV remodeling [10]. Also, study on the patients with advanced LV dysfunction (ejection fraction of less than 25%) shows that exercise training improves exercise capacity, has no adverse effect on ventricular remodeling, and does not cause serious cardiac complications [16]. Another study on patients with LV systolic dysfunction shows that 6-month exercise-based CR induced a combined reverse left atrial and LV remodeling as well as significant improvement in exercise functional capacity, LVEF, and early LV diastolic filling [17]. In contrast, one study on patients with Q wave MI showed that exercise training had adverse effect on ventricular asynergy and caused more shape distortion, expansion, and thinning in patients with 18% LV asynergy [11]. However, the exercise program used in their study seems not to be standard [18], and a review on 48 trials suggested that standard rehabilitation program is beneficial for all CAD patients [6]. The mechanism by which the exercise rehabilitation is beneficial for CAD patients has not been clarified yet. The described mechanisms for the effect of exercise on CAD patients are (a) improvement in endothelial function, autonomic tone, and myocardial oxygen demand, (b) modification of inflammatory markers, coagulation, and clotting factors, and (c) development of coronary collateral vessels [19, 20].

Our study had some limitations. Since we did not have control group we cannot estimate the exact effect of CR on CAD patients apart from the routine medical medication. Also, our study was not long enough to determine long-term results of CR in our patients. Therefore, controlled studies with longer follow-ups are needed in Iranian population to determine the exact effect of CR program on ventricular remodeling in CAD patients.

## 5. Conclusions

Cardiac rehabilitation in post-MI patients with LV dysfunction could have beneficial effects on cardiac function without adversely affecting LV remodeling or causing serious cardiac complications. Further well-designed trials with longer follow-ups are required in this regard.

## Figures and Tables

**Table 1:** Demographic data of the patients.

Age (years)	57.5 ± 10.24
Male/female	54/16 (77.1% M)
Hypertension	26 (37.3%)
Diabetes mellitus	24 (34.3%)
Hyperlipidemia	37 (52.9%)
Smoking	8 (11.4%)
Family history	47 (67.1%)
CABG	36 (51.4%)
PTCA	17 (24.3%)
Beta blockers	48 (68.5%)
Statins	56 (80.0%)
Aspirin	61 (87.14%)

Data are presented as the mean ± SD or number (%); CABG: coronary artery bypass grafting; PTCA: percutaneous transluminal coronary angioplasty.

**Table 2:** Echocardiographic data of patients before and after the rehabilitation period.

	Before CR	After CR	*P* value
LVEF (%)	45.14 ± 5.77	50.44 ± 8.70	<0.001
METs	8.00 ± 2.56	10.08 ± 3.00	<0.001
LVESD (mm)	38.91 ± 10.83	38.09 ± 9.04	0.378
LVEDD (mm)	54.63 ± 12.96	53.86 ± 8.95	0.529
Maximum heart rate	45.19 ± 98.13	56.17 ± 69.13	0.15
Test duration and recovery	20.5 ± 81.14	06.6 ± 17.15	0.57

Data are presented as mean ± SD. CR: cardiac rehabilitation; LVEF: left ventricular ejection fraction; METs: peak exercise capacity measured in Metabolic equivalents; LVESD: left ventricular end-systolic diameter; LVEDD: left ventricular end-diastolic diameter.
